# The clinical aspects of septo-optic dysplasia: A narrative review with illustrative case report

**DOI:** 10.1016/j.ijscr.2023.108575

**Published:** 2023-07-27

**Authors:** Mohammed Maan Al-Salihi, Tabarak Qassim, Narjiss Aji, Maryam Sabah Al-Jebur, Md Moshiur Rahman, Ali Ayyad

**Affiliations:** a Department of Neurosurgery, Hamad General Hospital, Doha, Qatar; b Royal College of Surgeons in Ireland, Bahrain; c Faculty of Medicine and Pharmacy of Rabat, Morocco; d College of Medicine, University of Baghdad, Baghdad, Iraq; e Neurosurgery Department, Holy Family Red Crescent Medical College, Dhaka, Bangladesh; f Department of Neurosurgery, Saarland University Hospital, Homburg, Germany; g Department of Neurological Surgery, School of Medicine and Public Health, University of Wisconsin, Madison, WI, USA

**Keywords:** Clinical features, De Morsier syndrome, Midline brain anomalies, Optic nerve hypoplasia, Septo-optic dysplasia, Case report

## Abstract

**Introduction and importance:**

Septo-optic dysplasia (SOD) is a rare congenital disorder characterized by abnormal development of the optic nerve, pituitary gland, hypothalamus, and midline brain structures, with heterogeneous presentation among cases.

**Case presentation:**

We report a seven-month-old male infant presented with persistent vomiting and delayed developmental milestones. He had dysmorphic facial features, bilateral esotropia, a head circumference of 50 cm, and scoliosis. His muscle tone was high (clasp-knife spasticity) and his deep tendon reflexes were brisk in the four limbs. Clinical evaluation and brain MRI confirmed the diagnosis of SOD, for which, he was subjected for multidisciplinary evaluation. Genetic testing revealed an autosomal dominant TUBB gene mutation. On follow-up, at the age of three years, he presented with recurrent focal motor and generalized seizures, which were controlled with levetiracetam.

**Clinical discussion:**

The ophthalmic manifestations of SOD include optic nerve hypoplasia, which can lead to visual impairments such as nystagmus, strabismus, and reduced visual acuity. Midline brain anomalies involve structures like the corpus callosum and septum pellucidum, and can result in cognitive and neurological deficits. Hypothalamic-pituitary axis abnormalities can cause endocrine dysfunction and growth abnormalities. The clinical heterogeneity of SOD is attributed to variable phenotypic penetration and genetic mutations. Environmental risk factors may also contribute to the development of the syndrome.

**Conclusion:**

SOD is a complex disorder with diverse clinical manifestations. Early diagnosis and multidisciplinary management are crucial for optimizing patient outcomes. Further research is needed to understand the underlying genetic and environmental factors involved in SOD and to develop targeted treatments.

## Highlights

• SOD is denoted by a triad of optic nerve hypoplasia, hypothalamicpituitary axis abnormalities & midline brain anomalies.
• The first case of septo-optic dysplasia was described in 1941 by David Reeves.
• The prognosis is highly variable between patients and depends on the constellation of symptoms.

## 1 Introduction

Septo-optic dysplasia (SOD) is a rare congenital disorder characterized by in-utero aberrant development of the optic nerve, optic chiasma, pituitary gland, hypothalamus, and/or midline structures (e.g., septum pellucidum, corpus callosum, and others) [1,2]. The disorder comprises wide heterogeneous abnormalities in the development of these structures varying from complete agenesis of any or a combination of these structures or abnormalities in their genesis (dysgenesis) [3,4].

Clinically, SOD is characterized by a triad of optic nerve hypoplasia, hypothalamic-pituitary axis dysfunction, and defect of the brain midline structures. At least two of the three features of this triad should exist to make the diagnosis of SOD [2]. To the best of our knowledge, this is the first SOD case in this age group to present with facial dysmorphism and TUBB gene mutation. Taking this into consideration, and in addition to the rare incidence of SOD diagnosis, its heterogeneous presentation, and wide variation in the severity of its clinical features, it compelled us to conduct a literature review to address SOD including its history, epidemiology, clinical presentation, diagnostic modalities, treatment, and prognosis. The case report has been prepared in line with the SCARE criteria [5].

## 2 History and syndrome nomenclature

The first case of SOD was described in 1941 when David Reeves noted an association between optic nerve hypoplasia and agenesis of the septum pellucidum in a four-month-old infant in Bull Johns Hopkins Hospital [6]. A decade later, a French pediatrician – George De Morsier – theorized that the two abnormalities are connected, coining the term ‘septo-optic dysplasia [7]. In 1970, Hoyt et al. described the three features of the SOD and named the syndrome ‘De Morsier’ syndrome after the French pediatrician's name [8]. Later on, several detailed case reports were published, but the SOD is still being commonly known as ‘De Morsier's syndrome’ given his initiative remarkable contribution to understand this syndrome [9].

## 3 Epidemiology

SOD is rare. In a population-based study in Northwest England, an incidence of 1 per 10,000 livebirths was reported in 2006 [10]. Data from the National Cooperative Growth Study (NCGS) reported a prevalence of 6.3 cases per 100,000 live births [11]. The syndrome occurs in both males and females equally, and it is more prevalent among infants born to younger mothers [1].

## 4 Etiology and pathogenesis

SOD is a congenital disorder of early brain development [3,4]. The constellation of symptoms that comprise this syndrome is presumed to result from failure of the early development forebrain (occurring at 4–6 weeks of gestation) [12]. This is the critical period of formation of the anterior neural plate which eventually results in the morphogenesis of the optic nerves, hypothalamic-pituitary axis, and midline brain structures [12]. The vast majority of the reported cases in the literature are non- hereditary [1,3,8,12]. However, a number of familial cases have been reported [13]. Accordingly, several etiological factors have been suggested, and a combination of genetic predisposition and environmental factors has been theorized [1]. The precise etiology, to date, remains elusive.

### 4.1 Genetic etiology

Several mutations in key developmental genes were identified and described in infants of SOD [13]. The most common of these genes are HESX homeobox 1 (HESX1), SRY-box transcription factor 2 (SOX2), and SRY-box transcription factor 3 (SOX3) [13]. Other reported genetic mutations include aryl hydrocarbon receptor nuclear translocator 2 (ARNT2), fibroblast growth factor receptor 1 (FGFR1), orthodenticle homeobox 2 (OTX2), and prokineticin receptor 2 (PROKR2) [13]. Both heterozygous and homozygous genetic mutations have been described [13,14]. Different modes of inheritance including autosomal dominant and autosomal recessive modes were identified [14]. Multigenetic etiologies were also reported [14].

The clinical phenotypic features of SOD have been reported to be caused by certain genetic mutations. For instance, the anophthalmia or microphthalmia has been reported in infants with SOX2 duplications or mutations (3q26.3-q27) [15]. Mutations and/or duplications of SOX3 gene (X26.3) have been implicated in the development of hypothalamic-pituitary axis abnormalities and midline brain anomalies [13,16]. Pituitary gland dysplasia and impaired endocrinal function have been reported in association with OTX2 gene mutations (14q21-q22) [17]. These genes are thought to be responsible for the embryonic development of the eyes, optic nerves, pituitary gland, and midline brain structures [13,14].

Within a single pedigree, the phenotypes and the penetrance are highly variable [14]. Therefore, the etiology of septo-optic syndrome is likely a complex interaction between genetic predisposition and environmental risk factors, rather than a mere genetic disease arising from known genetic mutations [14]. Genetic mutations are identified in <1 % of infants born with fibro-optic dysplasia [1,13,14].

### 4.2 Environmental risk factors

Seeing that the precise cause of septo-optic syndrome is unknown in the most of the cases, researchers hypothesized that a combination of genetic and environmental factors might play a role in the development of the syndrome [1,13]. Though the role of genetic mutation is crucial in the development of SOD, the contribution from environmental factors have also been suggested to play a role in the risk of development of the disease in sporadic cases [13].

Several environmental risk factors were proposed such as viral infections (e.g., cytomegalovirus), maternal insulin-dependent diabetes mellitus, maternal alcohol abuse, fetal alcohol syndrome, maternal intake of medications (such as quinine, antiepileptic drugs such as valproic acid, and illicit drugs as amphetamine and cocaine), maternal young age (usual age at conception or delivery <22 years), primigravida, and disruption in maternal-fetal circulation during critical periods of early brain development [18].

## 5 Clinical presentation

**Fig. 1:**
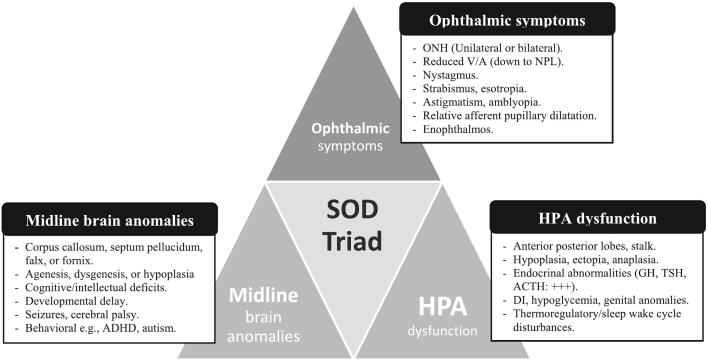
A summary of the triad of SOD and its clinical manifestations.

Patients with septo-optic syndrome usually manifest during the neonatal period, infancy, or early childhood. Their clinical features are highly variable but can be summarized under three major categories (as will be detailed in this section): ophthalmic manifestations, hypothalamic-pituitary axis abnormalities, and midline brain anomalies (Fig. 1).

**Fig. 2:**
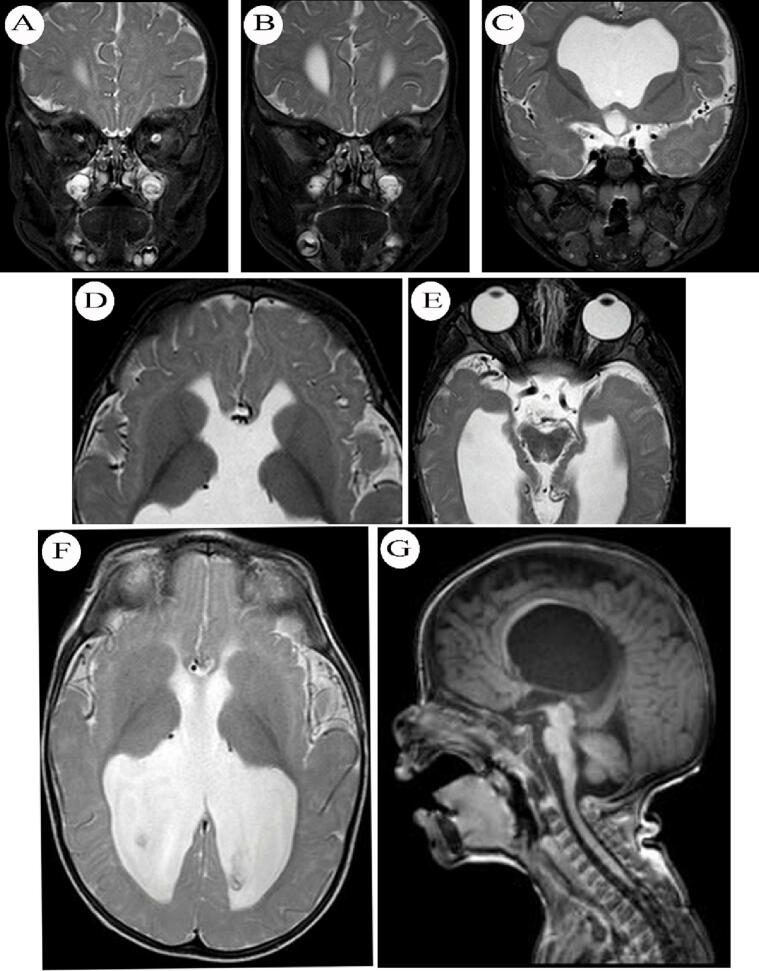
Brain MRI of the child presented in Box 1. Images A, B, and C are T2 coronal sections showing hypoplastic optic nerves and chiasma with a normal-sized pituitary gland. Image D is an axial T2 section showing box-like dilated frontal horns of the lateral ventricles. Image E is another T2 axial section showing hypoplastic optic nerves and chiasma and dilated lateral ventricles. Image F is an axial T2 film showing dilated lateral and third ventricles, colpocephaly, and absent septum pellucidum. Image G is a sagittal T1 section showing dilated lateral ventricle and thinned out hypoplastic corpus callosum.

It is to be noted that less than one third of the cases (∼30 %) present with the three features of the SOD triad [1]. The presence of one feature of the triad of SOD might suggest the possibility of the syndrome. However, at least two features of the triad are required to make a definitive diagnosis of SOD [2]. Moreover, the severity and the age at disease onset also vary [[1], [2], [3],^11^]. Some patients present with multiple congenital anomalies at birth, whereas others do not manifest except later in early childhood with mild cognitive or visual abnormalities [[1], [2], [3],^11^].

The clinical heterogeneity is largely attributed to the variability in phenotypic penetration [2]. Visual manifestations are the main reason for seeking medical advice, representing 60–90 % of presentations to the physicians [18]. Three-fourths of these patients have other associated neurological and endocrinological deficits [18].

### 5.1 Ophthalmic manifestations

Ophthalmic manifestations are usually the main cause of clinical presentations to the pediatric clinic [19]. Optic nerve hypoplasia (ONH) is the primary and major feature of SOD [20]. Optic nerve hypoplasia is unilateral in most of the cases (∼57 %). Clinically, ONH presents with nystagmus (if bilateral optic nerves were involved), strabismus (if unilateral optic nerve was affected), diminution of visual acuity (down to no light perception in approximately 23 % of the cases), esotropia, enophthalmos, amblyopia, and/or astigmatism [18,19]. Common signs encountered on clinical examination include relative afferent pupillary defect, optic disc pallor, absent fixation, visual inattentiveness, searching nystagmus, strabismus, and colobomas of the iris, choroids, and retina [19].

### 5.2 Midline brain anomalies

Anomalies of the midline brain structures, including corpus callosum, septum pellucidum, falx, and fornix, are the second feature of SOD occurring in approximately 60 % of the patients [20]. Anomalies of these structures range from complete agenesis to mild dysgenesis or hypoplasia [1,20]. Cortical anomalies were also described in patients with SOD, referred to as SOD-plus syndrome [21]. Commonly described cortical anomalies include hippocampus anomalies, cortical dysplasia, polymicrogyria, cortical atrophy, and schizencephaly [19].

Clinically, midline brain anomalies might be asymptomatic (i.e., incidental findings on brain imaging) or present with a wide range of neurological manifestations [[1], [2], [3],^6^]. Common neurological presentations of midline brain anomalies include cognitive/intellectual deficit, mental retardation, developmental delay, seizures, cerebral palsy, attention deficit disorder, and autism (see the case presented in Box 1) [[1], [2], [3],^6^]. It is worth mentioning that agenesis of septum pellucidum and/or corpus callosum are not associated with specific cognitive/intellectual deficits or certain developmental abnormalities [19,20]. In fact, the other features of SOD (i.e., visual impairment and endocrinal abnormalities) contribute significantly to the development and evolution of cognitive and developmental defects [4,6,8,19,20].

Box 1 **Case presentation**

A seven-month-old male infant was brought by his mother to the emergency department with persistent vomiting and delayed developmental milestones. He was unable to lift his head, roll over, or sit unsupported. He was born to non-consanguineous parents. The labor was a non-eventful spontaneous vaginal delivery. He was born at term (gestational age 38/40) with a low birth weight (2630 g). He had dysmorphic facial features, bilateral esotropia, a head circumference of 50 cm, and scoliosis. His muscle tone was high (clasp-knife spasticity) and his deep tendon reflexes were brisk in the four limbs.

During workup, a standard multiplanar brain MRI was performed (Fig. 2) and revealed absence of septum pellucidum (Fig. 2F) and dilatation of the lateral and third ventricles (Fig. 2F), with box-like configuration of the frontal horns of the lateral ventricles (Fig. 2D), and colpocephaly (Fig. 2F). There was no evidence of increased intracranial pressure or transependymal cerebrospinal fluid (CSF) seepage. The corpus callosum was stretched and thinned out, and the splenium was not clearly seen (possibly hypoplastic) (Fig. 2G). Optic nerves and chiasma were hypoplastic (Fig. 2A, B, and E). As for the hypothalamic-pituitary axis, there were no abnormalities to note; the pituitary gland, pituitary infundibulum, and olfactory nerves were within normal physiological limits (Fig. 2C). There was no evidence of neural migration, heterotopia, or polymicrogyria. Magnetic resonance imaging of the whole spine revealed mild scoliosis of the dorsolumbar spine, but the spinal cord, conus medullaris, and cauda equina were normal. He had autosomal dominant TUBB gene mutation on genetic testing. The patient was diagnosed with septo-optic dysplasia and was discharged for further multidisciplinary evaluation.

At the age of three years, he presented with recurrent focal motor and generalized seizures. His motor and developmental milestones were delayed. His best motor performance was to crawl and creep (he could not walk or stand). Though his hearing tests were within normal physiological limits, his speech and language milestones were poor. He could hardly use a few words. He was treated with levetiracetam to control his seizures and was referred to orthopedic department for further review. Case report followed the SCARE guidelines for its realization [5].

Alt-text: Box 1

### 5.3 Hypothalamic-pituitary axis abnormalities

Abnormalities of the hypothalamus and the pituitary gland are the third feature of the SOD triad [2]. Common reported abnormalities of the hypothalamic pituitary axis (HPA) include anterior pituitary hypoplasia, ectopic posterior pituitary gland, and thin or absent pituitary stalk [19,22]. Hypopituitarism was encountered in 62 % to 80 % of the cases of SOD [19]. Growth hormone deficiency is the most common described anomaly, presenting with short stature and developmental motor delay [23]. Deficiencies of thyroid stimulating hormone (TSH) and adrenocorticotrophic hormone (ACTH) are the next most common abnormalities described [19,22,23]. In rare cases, adrenal insufficiency and diabetes insipidus were reported [19]. Severe panhypopituitarism presenting with failure to thrive, hypoglycemia, genital anomalies, and abnormalities in sexual development was also described [22]. Accordingly, suspected cases of SOD should be assessed for the HPA dysfunction as early detection and treatment of endocrine deficits in such cases can be lifesaving.

Other manifestations of HPA dysfunction include childhood obesity, asymmetrical facial and limb bones development, voice changes, hypoglycemia, micro-penis, hypoplastic testes, cryptorchidism, precocious puberty, thermoregulatory abnormalities, and sleep wake cycle abnormalities [1,22,23].

### 5.4 Other clinical manifestations

The signs and symptoms of SOD are not confined to the previously described triad. Other several manifestations were described in association. Other associated developmental anomalies include cerebellar hypoplasia, underdeveloped midfacial structure, bilateral cleft lip/palate, high arched palate, flat nasal bridge, scoliosis (see the case presented in Box 1), cardiac and digital abnormalities, low Apgar scores, and failure to thrive, in addition to anosmia, sensorineural hearing loss, involuntary movements, hyperbilirubinemia, prolonged jaundice, hypotonia, lethargy, and irritability. The clinical presentation of SOD can vary significantly [1]. Some researchers suggest that SOD should actually be considered a group of heterogeneous related disorders rather than a single one [1]. The term septo-optic dysplasia spectrum is commonly used for this reason.

## 6 Differential diagnosis

The list of differential diagnosis of SOD is wide. Each of the major features of the syndrome can occur either separately or in the context of another syndrome [2]. A common example for this is the optic nerve hyperplasia which can occur in SOD or in the more common ONH syndrome [2]. Moreover, the variety of associated anomalies in SOD plus syndrome (e.g., hydrocephalus, heterotopia, and polymicrogyria) adds to the complexity of differential diagnosis [21]. Congenital hypopituitarism and holoprosencephaly should be considered in the differential diagnosis list of SOD [4,22,23].

## 7 Diagnosis

**Fig. 3:**
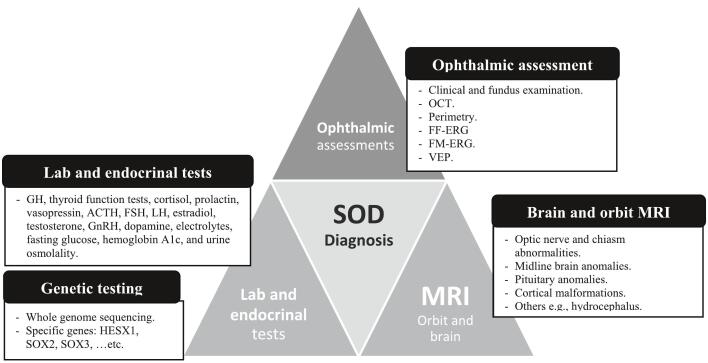
An overview of the diagnostic tools for SOD.

The diagnosis of SOD is mainly clinical. However, several investigations aid in confirming the diagnosis. These investigations include ophthalmological studies, magnetic resonance imaging (MRI), endocrinal tests, genetic studies, and other laboratory tests (Fig. 3).

### 7.1 Ophthalmic studies

Several ophthalmic assessments can be used to diagnose SOD. Initial clinical examination is crucial to assess the degree of visual acuity, pupillary light reflex, and the presence of strabismus, nystagmus, esotropia, microphthalmia, coloboma, abnormal fixation, abnormalities of saccadic movements, or other signs [[1], [2], [3]]. Fundus examination is indicated to search for signs of optic nerve hypoplasia such as optic disc pallor, optic disc tilting, double-ring sign, tortuosity of the optic nerve, small optic disc, or small neuro-retinal rim area [[1], [2], [3]].

To confirm clinically observed ophthalmic abnormalities, several investigations can be utilized such as optic coherence tomography (OCT), perimetry, full field electroretinography (FF-ERG), focal macular ERG (FM-ERG), and visual evoked potential (VEP) [19]. Optic coherence tomography can be used to examine the thickness of ganglion cell layer and the estimated number of their axons [19]. Perimetry is used to detect visual field abnormalities commonly encountered in SOD such as bitemporal hemianopia, generalized constricted field, central scotoma, binasal scotoma, or altitudinal defects [19]. Electroretinography is beneficial in assessing the amplitude, latency, and photic responses of the retina. Visual evoked potential can detect delayed latency along the optic nerves [19].

### 7.2 Magnetic resonance imaging

Brain and orbit MRI has a good yield. It shows abnormalities in the optic nerve and chiasma, midline brain structures, hypothalamic pituitary axis, cortical malformation, or other findings [1,3].

Typical anomalies of the optic pathway include optic nerve hypoplasia (either unilateral or bilateral), optic chiasma hypoplasia, optic chiasma agenesis, or non-decussating retinal fugal fibers [1,4].

Common midline brain anomalies include agenesis or thinning of the corpus callosum, agenesis or hypo genesis of the septum pellucidum, cavum septum pellucidum, hypoplasia of falx, and hypoplasia of fornix [1,20].

Pituitary abnormalities encountered in SOD include hypoplastic anterior pituitary, ectopic posterior pituitary, thin pituitary stalk, or agenesis of the pituitary stalk. Cortical malformations are not uncommon in SOD such as polymicrogyria, cortical focal dysplasia, cortical atrophy, Schizencephaly, and hippocampus anomalies [21].

Hydrocephalus, Chiari I malformation, cerebellar hypoplasia, and arachnoid cyst can also occur in patients with SOD [1,20].

### 7.3 Endocrinal tests

Laboratory and endocrinal tests are also an integral part in diagnosing SOD [22]. A full hormonal panel should be requested including somatrophic tests (e.g., growth hormone and insulin growth factors), thyroid function tests (i.e., thyroid stimulating hormone (TSH), free T3, and free T4), cortisol tests (e.g., ACTH), prolactin level, dopamine level, gonadotropin function tests (e.g., follicular stimulating hormone (FSH), luteinizing hormone (LH), estradiol, testosterone, and gonadotropin releasing hormone (GnRH)), vasopressin, electrolytes, fasting glucose, hemoglobin A1c, and urine osmolality [1,22,23]. The tests should be performed on regular basis, at least every six months [1].

### 7.4 Genetic studies

Though genetic abnormalities are encountered in <1 % of all cases of SOD, establishing the genotype is essential for making an accurate early diagnosis, understanding the disease etiology, proper management, and providing proper genetic counseling [13,14].

Both whole exome sequencing and specific gene testing might be used in suspected cases of SOD. Common genetic mutations include HESX1, SOX2, SOX3, ARNT2, FGFR1, OTX2, and PROKR2 [13].

### 7.5 Additional investigations

Depending on clinical presentation additional investigations might be required on a case-by-case assessment based on their clinical presentation. For instance, electroencephalography (EEG) is indicated in cases presenting with seizures [1]. Neuropsychological tests are required for children with autism or ADHD [18]. Polysomnography can be used in patients with sleep wake cycle problems. Skeletal abnormalities warrant the use of plain X-ray or MRI of the affected skeleton [18].

## 8 Antenatal diagnosis

The diagnosis of SOD can be made in the antenatal period [1,8]. This is made based on fetal ultrasonography (US), fetal MRI, and genetic amniocentesis. Termination of pregnancy can be carried out if decided [2,3]. Genetic counseling can also be provided to families where mutations have been identified.

## 9 Management

The mainstay management strategy of SOD is symptomatic treatment [3]. Multidisciplinary approach is crucial in the management of children with SOD, and regular follow-up (optimally every six-month) should be encouraged [19]. Lifelong monitoring is recommended in some experts' opinions [19]. Visual interventions can be used for improvement of strabismus or refractive errors [19]. However, the remaining optical and visual anomalies are not treatable. Physical therapy can improve limb spasticity. Hormonal replacement should be considered in patients with hormonal deficiencies, particularly growth and thyroid hormones deficiency [20,22]. A balanced diet and weight control programs help children with obesity [24]. Occupational therapy, speech therapy, and psychological support may be required for rehabilitation [19].

Regular follow-up of patients with SOD is essential to early identify evolving hormonal deficiencies and developmental delay [1]. Early initiation and optimization of hormonal replacement therapy can improve the patient's neurodevelopmental, motor, and cognitive deficits [20].

## 10 Prognosis

The prognosis is highly variable between patients and depends on the constellation of symptoms compromising the syndrome and their severity [2,3]. No cure exists for SOD [3]. However, the outcome of the disease can be optimized with appropriate symptomatic treatment [19]. For instance, early and adequate correction of growth hormone deficiency help children to achieve heights approximately similar to their counterparts from the general population [19]. Similarly, early correction of hypothyroidism optimizes the motor and neurocognitive performance of the children [8]. Adequate control and secondary prevention of seizures, hypoglycemic events, and thermoregulatory abnormalities improves long-term prognosis and survival [2,3].

## 11 Conclusion

SOD is a rare congenital disease characterized by a triad of optic nerve hypoplasia, hypothalamic pituitary axis abnormalities, and midline brain anomalies. The diagnosis is clinically made, but several investigations can help in confirming the diagnosis. The mainstay management is symptomatic treatment, and the prognosis depends on the clinical features and their severity.

## Patient consent

Written informed consent was obtained from the patient's parents/legal guardian for publication and any accompanying images. A copy of the written consent is available for review by the Editor-in-Chief of this journal on request.

## Ethical approval

Giving that the approach was used as a standard treatment for these conditions in the hospital, the retrospective data are anonymized, no risks of patient's recognitions exist and no experiments were done, ethical approval was not required to use the patients' anonymous data for research purposes. Thus, the Hamad General hospital waved the need for the ethical approval.

## Funding

There were no sponsors for this case report.

## Registration of research studies

N/A.

## CRediT authorship contribution statement

All authors equally contributed to the analysis and writing of the manuscript.

## Declaration of competing interest

The authors declare they have no conflicts of interest.
